# Dynamic analysis of growth characteristics, secondary metabolites accumulation, and an in-depth understanding of anthraquinones biosynthesis in *Rubia cordifolia* Linn.

**DOI:** 10.3389/fpls.2024.1504863

**Published:** 2025-01-07

**Authors:** Conglong Lian, Xiuyu Liu, Kaihua Guo, Hao Yang, Jingfan Yang, Jinxu Lan, Suiqing Chen

**Affiliations:** ^1^ School of Pharmacy, Henan University of Chinese Medicine, Zhengzhou, China; ^2^ Henan Key Laboratory of Chinese Medicine Resources and Chemistry, Henan University of Chinese Medicine, Zhengzhou, China; ^3^ Collaborative Innovation Center of Research and Development on the Whole Industry Chain of Yu-Yao, Henan University of Chinese Medicine, Zhengzhou, Henan, China; ^4^ Co-Construction Collaborative Innovation Centre for Chinese Medicine and Respiratory Diseases by Henan & Education Ministry of China, Henan University of Chinese Medicine, Zhengzhou, China

**Keywords:** *Rubia cordifolia*, growth characteristic, secondary metabolites, SMRT sequencing, anthraquinones biosynthesis

## Abstract

*Rubia cordifolia* is a well-known plant used in oriental medicine plant, and is also serves as the primary traditional source of plant red dyestuffs. With the current depletion of natural resources of *R. cordifolia*, it is critical to conduct cultivation studies on the *R. cordifolia*. Here, we report on the dynamic growth characteristics and secondary metabolite accumulation of cultivated *R. cordifolia*, as well as the discovery of important genes involved in anthraquinone biosynthesis. The results showed that *R. cordifolia* grows better in sunny environments than in shaded environments, and its triennials better than its biennials, base on the biomass and the concentration of the primary components purpurin and mollugin. The dynamic accumulation of purpurin and mollugin content suggested that 30 June to 15 October is a fair window for harvesting *R. cordifolia*, and the possibility of a specific transition connection during the purpurin and mollugin biosynthesis process. Furthermore, we sequenced *R. cordifolia* using SMRT technology for the first time and obtained 45,925 full-length transcripts, 564 alternative splicing events, 3182 transcription factors, 6454 SSRs, and 6361 lncRNAs. We hypothesized an anthraquinone biosynthetic pathway and found 280 full-length transcripts that may be involved in anthraquinone biosynthesis in *R. cordifolia*. In addition, RT-qPCR was used to detect the relative expression levels of 12 candidate ungenes in the above- and underground parts of *R. cordifolia*. Above all, our findings have crucial implications for the field management of cultivation and harvesting of cultivated *R. cordifolia*, and also provide useful genetic information for clarifying the potential genes involved in anthraquinone biosynthesis.

## 1 Introduction

*Rubia cordifolia* L. is a perennial climbing or scrambling herb plant with red rhizomatous base and roots belonging to Rubiaceae family. There stems often with much branched, quadrangular, with ribs rounded to thinly winged, sparsely to densely retrorsely aculeolate. Leaves in whorls of 4 or more (up to 8 or rarely 12). *R. cordifolia* is widely distributed from E and SE Asia to Afghanistan, from Sudan to S Africa (Flora of China Editorial Committee, 2006). Its dried roots and rhizomes are used in medicine, and the traditional Chinese medicine name is called RUBIAE RADIX ET RHIZOMA (Committee, 2020). *R. cordifolia* has been used in medicine for thousands of years. It was first published in the “*Shennong’s Herbal Classic*” during the Qin and Han Dynasties and was classified as a high-quality product (Ma, 2013). *R. cordifolia* is mainly used to treat hematemesis, epistaxis, hemorrhage, trauma bleeding, stasis of amenorrhea, joint arthralgia pain, swelling, and pain relief. It is commonly used in clinical practice as a Chinese medicine (Committee, 2020; Wen et al., 2022). Over the past few decades, more than a hundred compounds have been discovered in *R. cordifolia*, including anthraquinone, naphthoquinone, cyclohexepeptides, terpenes, polysaccharides, and other compounds (Son et al., 2008; Itokawa et al., 1993; Rao et al., 2006; Wen et al., 2022). Purpurin and mollugin are the two main active ingredients specified in the “*Chinese Pharmacopoeia”*, and serve as the benchmark components for assessing the quality of *R. cordifolia* (Committee, 2020). Among them, purpurin belongs to the anthraquinone group, and has the potential to exhibit anti-cancer, anti-microbial, antigen toxicity, and neuroregulatory effects (Kwon et al., 2023; Singh et al., 2021). Mollugin belongs to the naphthoquinone group, and plays an important role in anti-cancer, and anti-inflammatory properties (Ke et al., 2022; Li XR et al., 2023; Wang et al., 2023b). With the advancement of pharmacological research, the range of pharmacological activities of *R. cordifolia* has been continuously expanded. These activities include antioxidant, neuroprotective, anti-inflammatory, liver-protective, anti-rheumatic, hemostatic, anti-tumor, and immune regulation functions (Wen et al., 2022). In addition, *R. cordifolia* is also a well-known source of red plant dyestuffs that can be used for dying clothing and food (Vankar et al., 2008).

In the past, the source of *R. cordifolia* was mainly from wild resources. As the medicinal value of *R. cordifolia* continues to be explored, the market demand for this plant is increasing. However, due to the continuous reduction of wild resources, the supply of wild *R. cordifolia* can no longer meet the market demand. Furthermore, the quality of wild *R. cordifolia* is highly variable due to its geographical distribution, which makes it difficult to control and affects its clinical application. According to the Chinese Pharmacopoeia, the purpurin content should not be less than 0.1%, and the mollugin content should not be less than 0.4% (Committee, 2020). The previous investigation of the *R. cordifolia* market revealed several substandard phenomena. Previous studies have shown that the content of key ingredients in Chinese medicinal plants undergoes significant changes during different stages of growth and development. Moreover, the content of these key ingredients varies during different harvest periods, which directly affects the quality and efficacy of medicinal materials (Li and Wu, 2018). Therefore, it is essential to engage in large-scale cultivation and planting of *R. cordifolia* to ensure high-quality, controllable, and sustainable development of the plant. However, the cultivation technology and research on *R. cordifolia* are still at an early stage. Therefore, it is of great significance to enhance and regulate the quality of *R. cordifolia* by studying the growth characteristics and, more importantly, the accumulation of secondary metabolites in cultivated *R. cordifolia*.

In recent years, the investigation of essential genes involved in the biosynthesis of primary active compounds in Chinese medicinal materials has become a significant focus of research. This exploration represents a crucial aspect of the later-stage production of active ingredients from Chinese medicinal materials through synthetic biology (Yang et al., 2013). Purpurin, the primary active ingredient of *R. cordifolia*, belongs to the anthraquinone group, and the metabolic pathway of its biosynthesis has been less studied. Recent advances in sequencing technology have facilitated genome and transcriptome studies in many species, particularly with the development of third-generation single-molecule real-time (SMRT) sequencing technology. This technology can produce long reads up to 20 kb and bypass the assembly process, making it ideal for transcript recovery and isoform detection in well-sequenced and/or incomplete genomic sequences (Roberts et al., 2013).These advantages make SMRT an outstanding option for characterizing full-length transcripts. SMRT sequencing has been widely applied to many medicinal plant species, including *Salvia miltiorrhiza* (Xu et al., 2015), *Artemisia argyi* (Cui et al., 2021), and *Astragalus membranaceus* (Li et al., 2017). This technology has provided thousands of high-quality full-length transcripts and SSRs, and has also facilitated the identification of alternative splicing. However, genome and transcriptome sequencing in *R. cordifolia* has lagged behind that in other species, and there is limited information on the sequence and structure of its genes. Therefore, the generation of SMRT transcriptome data can provide valuable genetic information for the stufy of *R. cordifolia*.

In this study, we examined the growth characteristics, including the dynamic growth and biomass of the aboveground and underground parts, as well as the dynamic accumulation of metabolites such as purpurin and mollugin. Additionally, we constructed full-length cDNA libraries and analyzed them using SMRT sequencing technology for *R. cordifolia*. Furthermore, based on the obtained transcripts, coding sequences (CDS), long non-coding RNAs (lncRNA), transcription factors (TF), SSR analysis, and functional annotation using Gene Ontology (GO) and Kyoto Encyclopedia of Genes and Genomes (KEGG) were performed. In addition, the genes related to the biosynthesis of anthraquinones were identified based on KEGG annotation, and the expression of some genes was verified by RT-qPCR. In conclusion, this is the first comprehensive report to characterize the growth, composition accumulation, and the full-length transcriptome of *R. cordifolia* using SMRT sequencing. This study provided important guidance for the cultivation of *R. cordifolia* and laid a solid foundation for further research on the functional genes involved in the biosynthesis of active ingredients of *R. cordifolia*.

## 2 Materials and methods

### 2.1 The planting of *R. cordifolia*

*R. cordifolia* was planted in an experimental field located in the Henan Medicinal Botanical Garden of Henan University of Chinese Medicine (113°63’15’’ E, 34°74’99’’ N, 107 m). *R. cordifolia* was identified by prof. Suiqing Chen, and the voucher specimen with the number of 410046LY0088 was deposited in the specimen room of the School of Pharmacy, Henan University of Traditional Chinese Medicine. This region has a warm temperate continental monsoon climate, and the annual climatic conditions were as follows: The average annual pressure is 1,003.5 hPa; The average annual relative humidity is 52%; The average annual evaporation is 1,112.6 mm; The annual precipitation is 613.9 mm, with the highest precipitation occurring in July and August, reaching 285.1 mm; The annual sunshine duration is about 2,400 hours, with the longest average sunshine duration of 14 hours in June and the shortest of 9 hours in December; The average maximum temperature in July is 37.1°C, with the highest recorded temperature in the last five years reaching 41°C, and the lowest reaching -11°C. The average temperature from winter is around 0°C, and frost typically occurs from late December to mid-February. The experimental field was divided into three trial plots (TPs). TP1 (5×4 m^2^) and TP3 (4.5×3 m^2^) were located in a sunny environment with no trees blocking the sun. TP2 (12×2 m^2^) is located directly under pine and locust trees, lacks sunlight, and belongs to a shaded environment.

*R. Cordifolia* seeds were collected from Song County, Henan Province, China. Before sowing, the seeds were soaked in water at 25°C for 24 hours. TP1 and TP2 were sown in April 2020, while TP3 was sown in March 2021. The *R. cordifolia* plants were watered regularly, weeded only at the seedling stage, and no fertilizer was added throughout their life.

### 2.2 Sample collection and measurement

*R. cordifolia* from TP1 was selected to observe its phenological period from February 2021 to December 2022. For the detection of growth characteristics, *R. cordifolia* was harvested twice a month from 15 April, 2022, to 31 October, 2022. For the detection of secondary metabolite accumulation, *R. cordifolia* was harvested twice a month from 15 April, 2022, to 31 December, 2022. Samples were collected using a five-point sampling method for each plot, with six plants taken from each point. The samples were immediately placed in an incubator containing some water at room temperature, which is mainly used to moisturize and prevent wilting of the plant after harvest, and then taken back to the laboratory within one hour to measure the growth index, which includes the length and diameter of the primary root and stem. After quick rinse with running water to remove any remaining soil, the samples were washed with deionized water and dried with absorbent paper. The fresh weight, dry weight, and water content of both above- and underground parts were then measured. Furthermore, the remaining samples were placed on a testing bench to dry in order to detect the levels of purpurin and mollugin.

For the full-length transcriptome samples, tissue samples from the root, stem, leaf, and fruit were collected at the fruit stage. For RT-qPCR analysis, samples of both aboveground and underground parts were collected. The samples were wrapped in silver paper, exposed to liquid nitrogen for 15 minutes, and then transferred to an ultra-low-temperature refrigerator.

### 2.3 Determination of purpurin and mollugin concentrations

Chromatographic conditions: According to the Chinese Pharmacopoeia guidelines, the chromatography was performed on a Spherisob C18 column (250 mm × 4.6 mm, 5 μm) with octadecylsilane bonded silica gel as the filler. A mobile phase of methanol-acetonitrile-0.2% phosphoric acid solution (25:50:25) was used, and iso-elution was performed. Flow rate: 0.8 mL/min; Column temperature: 25°C; The detection wavelength is 250 nm; The sample size was 20 μL for the test product and 10 μL for the control product. The theoretical plate number calculated based on the peaks of purpurin and mollugin should not be less than 4,000.

Preparation of standard solution: Purpurin and mollugin standards were obtained by accurately weighing and adding methanol to create a solution containing 0.1 mg of mollugin and 40 μg of purpurin per 1 mL. Purpurin (No. wkq20022106) and mollugin (No. wkq20092801) with a purity of ≥98% by HPLC were purchased from Sichuan Veqi Biotechnology Co., LTD.

Preparation of the test product solution involved drying the samples at 60°C in a blast drying oven, grinding and sieving them (<850 μm), accurately weighing them to 0.5 g, placing them in a tapered bottle with a plug, adding 100 mL of methanol, sealing the bottle, and leaving it overnight. The solution was then subjected to ultrasonic treatment (power 250 W, frequency 40 kHz) for 30 minutes, followed by cooling and reweighing. Add methanol to make up for the lost weight, shake well, filter, and accurately measure 50 mL of the filtrate. After drying with steam, dissolve the residue by adding 20 mL of a 4:1 mixture of methanol and 25% hydrochloric acid. The solution was heated and hydrolyzed in a water bath for 30 minutes. It was then cooled immediately, and 3 mL of triethylamine was added. The mixture was stirred well. Then, 25 mL of the mixed solution was transferred to a measuring flask. Finally, methanol was added to the solution, thoroughly shaken, and then filtered. The filtrate obtained was used for subsequent analysis.

### 2.4 RNA extraction, SMRT full-length transcript library construction, and sequencing

For the PacBio SMRT full-length transcript library construction and sequencing, total RNA was extracted using Trizol reagent (Invitrogen, CA, USA) following the manufacturer’s instructions. The quantity and purity of total RNA were analyzed using the Agilent 2100 Bioanalyzer (Agilent, CA, USA), with a RIN number of 7 to less than 10. Subsequently, 5 μg of total RNA from different individual samples described above were pooled in equal quantities following the manufacturer’s instructions. The construction of libraries proceeded as follows: (1) The NEBNext Single Cell/Low Input cDNA Synthesis & Amplification Module was utilized to synthesize the full-length cDNA from mRNA; (2) PCR amplification of full-length cDNA; (3) Repair of full-length cDNA damage/terminal repair; (4) Connection of SMRT dumbbell connector. Subsequently, the constructed libraries were tested, and the qualified libraries were sequenced using primers F: GCAATGAAGTCGCAGGGTTG and R: AAGCAGTGGTATCAACGCAGAGT on the PacBio platform (Pacific Biosciences, Menlo Park, CA, USA).

After obtaining the raw data, the Iso-Seq3 pipeline (https://github.com/PacificBiosciences/IsoSeq) was used to analyze the raw sub-reads. This included the generation of circular consensus (CCS) sub-reads, classification of full-length reads, and clustering of full-length non-chimeric (FLNC) reads. After removing redundant sequences using CD-HIT software (with an identity threshold of > 0.99), the integrity of the non-redundant transcriptome was evaluated using BUSCO (Simao et al., 2015). The final transcript sequences can be directly used for subsequent bioinformatic analysis, such as homologous gene analysis, gene family classification, SSR detection, and alternative splicing, as well as lncRNA identification.

### 2.5 Detection of SSRs, CDSs, TFs, alternative splicing events, and lncRNAs

Simple sequence repeats (SSRs) in the transcriptome were identified using MISA (http://pgrc.ipk-gatersleben.de/misa/). Only transcripts with a size of ≥500 bp were used for SSR detection. Candidate coding regions were identified using TransDecoder (https://github.com/TransDecoder/TransDecoder/releases). Transcription factors (TFs) were predicted from the putative protein sequences using the iTAK software (Zheng et al., 2016).

For alternative splice prediction, we directly used Iso-SeqTM data to conduct all-vs-all BLAST with high identity settings. BLAST alignments that met all criteria were considered to be products of candidate alternative splicing (AS) events. Specifically, there should be two high-scoring segment pairs (HSPs) in the alignment, and both HSPs should have the same forward/reverse direction. Within the same alignment, one sequence should be continuous or have a small “overlap” size (less than 5 bp), while the other should be distinct to show an “AS gap”. The continuous sequence should align almost completely to the distinct sequence. The AS gap should be larger than 100 bp and at least 100 bp away from the 3’ or 5’ end. In addition, for lncRNA analysis, four computational analysis tools (CPC, CNCI, CPAT, and Pfam) were combined to distinguish non-protein coding RNA candidates from putative protein-coding RNAs in the transcripts. Transcripts longer than 200 nucleotides and containing more than two exons were selected as potential long non-coding RNA (lncRNA) candidates. Transcripts with coding potential were filtered out to obtain predicted lncRNA.

### 2.6 Functional annotation

Gene function was annotated using the DIAMOND software by searching the following databases: NR (NCBI non-redundant protein sequences), KOG/COG/eggNOG (Clusters of Orthologous Groups of proteins), Swiss-Prot (a manually annotated and reviewed protein sequence database), and KEGG (Kyoto Encyclopedia of Genes and Genomes). Furthermore, the InterPro integrated database of the InterProScan software was used to analyze the Gene Ontology (GO) orthology of the non-redundant transcript sequence. After predicting the amino acid sequence of the non-redundant transcript, HMMER software was used to compare it with the Pfam (Protein family) database to obtain annotation information for the transcript.

### 2.7 Gene expression analysis using quantitative reverse transcription polymerase chain reaction

To confirm the putative results of genes involved in anthraquinone biosynthesis, twelve unigenes from the four pathways, including the shikimic acid pathway, TCA cycle, MVA pathway, and MEP pathway, were randomly selected for RT-qPCR analysis. Samples of mixed aboveground and underground parts were collected, and RNA was isolated from them using Trizol reagent according to the manufacturer’s instructions. Total RNA from each *R. cordifolia* sample was reverse-transcribed using the BCS HIScriptTM All-in-One Mix with dsDNA. This kit was used to eliminate genomic DNA contamination and to synthesize the initial strand of cDNA through reverse transcription. The cDNA obtained was adjusted to the appropriate concentration based on quantification using an ultra-micro spectrophotometer and stored at -20°C for future use. The cDNA from *R. cordifolia* samples was used as templates, and the *18S* gene (Shkryl et al., 2011; Wang et al., 2023c) was served as the internal reference gene for RT-qPCR. The reaction system was as follows: 10 μL of 2 × TransStart Top Green qPCR SuperMix, 0.4 μL of 50 × Passive Reference Dye II, 0.4 μL each of forward and reverse primers (10 μM), 2 μL of sample cDNA, and 6.8 μL of ddH_2_O. 45 reaction cycles were completed under the following conditions: 94°C 30 s, 94°C 5 s, and 60°C 30s. Each RT-qPCR was performed with three biological replicates and three technical repetitions. The relative expression of genes was calculated using the 2^−ΔΔCt^ method (Livak and Schmittgen, 2001). RT-qPCR primers, and amplification efficiency are listed in **Supplementary Table 1**.

### 2.8 Statistical analysis

All data, including three biological replicates and three technical repetitions, were subjected to SPSS Statistics and Excel for analysis. GraphPad Prism 8.0.2 software was used to scientific illustration. Student’s t-test was used to detect the significant differences between individual means. Differences at the 1% level were considered significant and marked with an asterisk between different groups.

## 3 Results

### 3.1 Observation of the phenological period of *R. cordifolia*

**Figure 1:**
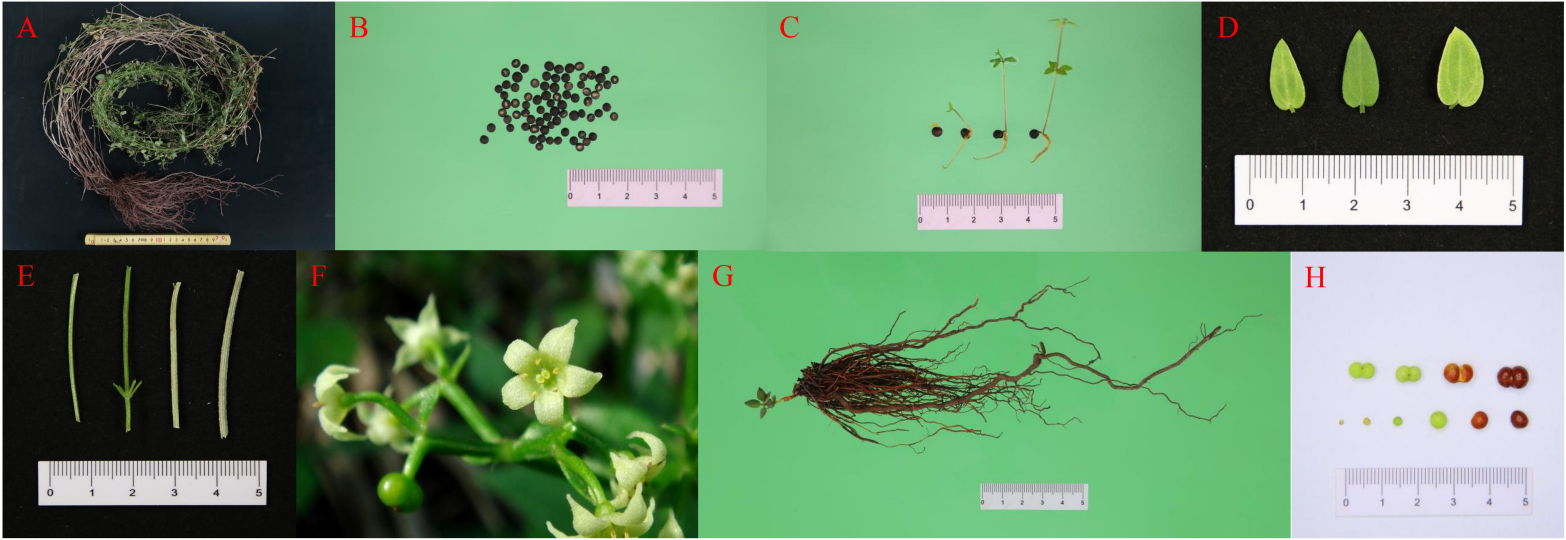
Different tissues of *R. cordifolia*. **(A)** Whole plant of *R. cordifolia*. **(B)** Seed. **(C)** Seedling. **(D)** Leaf. **(E)** Stem. **(F)** Flower. **(G)** Root. **(H)** Fruit.

During the period from February 2021 to December 2022, we continuously observed the growth and development of cultivated *R. cordifolia* plants. In 2021, as the temperature increased, so does the temperature of the soil, and the *R. cordifolia* seeds begin to germinate began on 28 March, the leaf-spreading period began on 7 April. With the accumulation of the effective cumulative temperature, the development of the floral organs was promoted, the bud stage commenced on 24 August, and the flowering period began on 16 September and lasted about for 35 days. With the arrival of winter, the temperature began to fall and the fruit began to ripen from 20 October to 7 November. and the fruit began to drop by 14 November. By 2 December, as the temperature continued to drop, more than half of the stems and leaves of the whole plant had turned yellow. Subsequently, the above-ground part dried up and completed its life cycle, which lasted about 250 days (**Figure 1**; **Table 1**). Vegetative growth lasted for 150 days, constituting approximately two-thirds of the life cycle, while reproductive growth lasted for 83 days, making up about one-third of the life cycle. On 23 February, 2022, with the arrival of spring and the revival of the earth, it was observed that the above-ground stem or underground rhizome of *R. cordifolia* began to sprout new buds, entering a new round of growth similar to the growth phenological period of 2021.

**Table 1:** Phenological period observation of *R. cordifolia*.

	2021 year	2022 year
Germination stage	March 28^th^	February 23^rd^
Leafing stage	April 7^th^	March 25^th^
Budding stage	August 24^th^	August 20^th^
efflorescence	September 16^th^	September 16^th^
Fruit ripeness stages	October 20^th^	October 16^th^
Fruit dropping stage	November 14^th^	November 18^th^
The withering stage	December 2^nd^	December 10^th^

### 3.2 Growth characteristics of *R. cordifolia*

**Figure 2:**
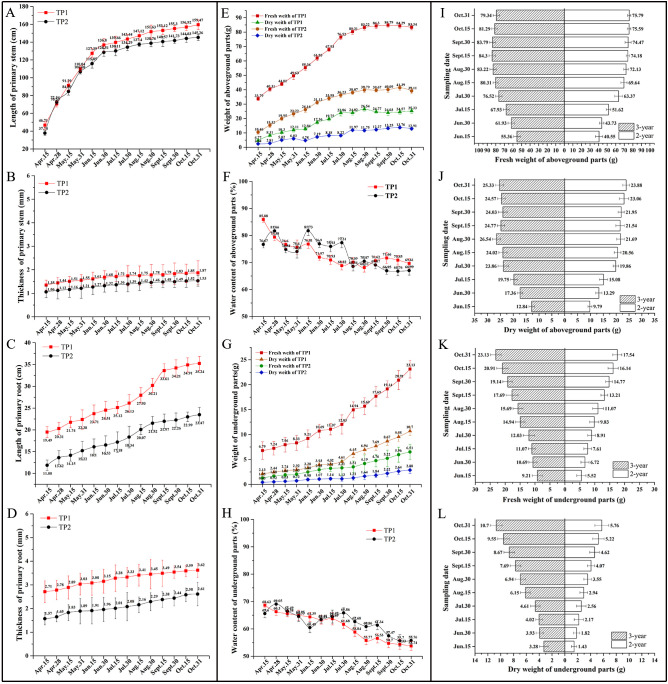
The dynamics of the growth characteristics of *R. cordifolia*. **(A)** Length of primary stem; **(B)** Thickness of primary stem; **(C)** Length of primary root; **(D)** Thickness of primary root; **(E)** Fresh and dry weight of aboveground parts of *R. cordifolia* growth in TP1 and TP2; **(F)** Water content of aboveground parts of *R. cordifolia* growth in TP1 and TP2; **(G)** Fresh and dry weight of underground parts of *R. cordifolia* growth in TP1 and TP2; **(H)** Water content of underground parts of *R. cordifolia* growth in TP1 and TP2; **(I)** Comparative analysis of fresh weight of aboveground parts of 2 and 3 year old *R. cordifolia*; **(J)** Comparative analysis of dry weight of aboveground parts of 2 and 3 year old *R. cordifolia*; **(K)** Comparative analysis of fresh weight of underground parts of 2 and 3 year old *R. cordifolia*; **(L)** Comparative analysis of dry weight of underground parts of 2 and 3 year old *R. cordifolia*.

The study of the dynamic growth and development of *R. cordifolia* stems revealed that the length and thickness of the stems grew rapidly from April to June, and then entered a period of slow growth from July to October (**Figures 2A, B**). The results for fresh weight and dry weight also exhibited a similar trend (**Figures 2E, G**). When comparing the growth of *R. cordifolia* in TP1 and TP2, it was observed that the *R. cordifolia* in TP1 exhibited better growth than that in TP2 (**Figures 2A, B, E**). The above results indicate that the growth of the above-ground part of *R. cordifolia* was significantly influenced by the environment. A sunny environment was found to support better photosynthesis, which in turn promoted the growth of the above-ground part of *R. cordifolia*.

Furthermore, the growth status of the underground part of *R. cordifolia* was also analyzed. The results showed that the length and thickness of the root of *R. cordifolia* exhibited an overall increasing trend. Specifically, the root length grew relatively fast in August, while the root thickness grew relatively slowly and steadily from April to October (**Figures 2C, D**). Examination of fresh weight and dry weight showed that the growth rate increased gradually from April to July and then accelerated from August to October (**Figure 2G**). When comparing the growth of *R. cordifolia* in TP1 and TP2, it was observed that the underground growth of *R. cordifolia* in TP1 was superior to that in TP2 (**Figures 2C, D, G**). Furthermore, the water content of both the above-ground and underground parts was also analyzed. The results indicated a decreasing trend in the water content of both the above-ground and underground parts as *R. cordifolia* grew from April to October (**Figures 2F, H**).

Finally, we compared the biomass of biennial and triennial *R. cordifolia* plants (**Figures 2I–L**). The results indicated that the overall trend of dynamic change in fresh weight and dry weight in the aboveground and underground parts of triennial and biennial plants was similar. There was an upward trend from June, and the bioaccumulation in the aboveground and underground parts of triennial plants was higher than that of biennial plants. The above-ground dry weight of the triennial and biennial *R. cordifolia* was 223.07g and 190.70g, respectively. The underground dry weight of the triennial and biennial *R. cordifolia* was 65.55g and 34.14g, respectively. The results showed that the growth of the triennial *R. cordifolia* was significantly better than that of the biennial *R. cordifolia*, especially in terms of root biomass.

### 3.3 Dynamic accumulation of active ingredients in *R. cordifolia*

**Figure 3:**
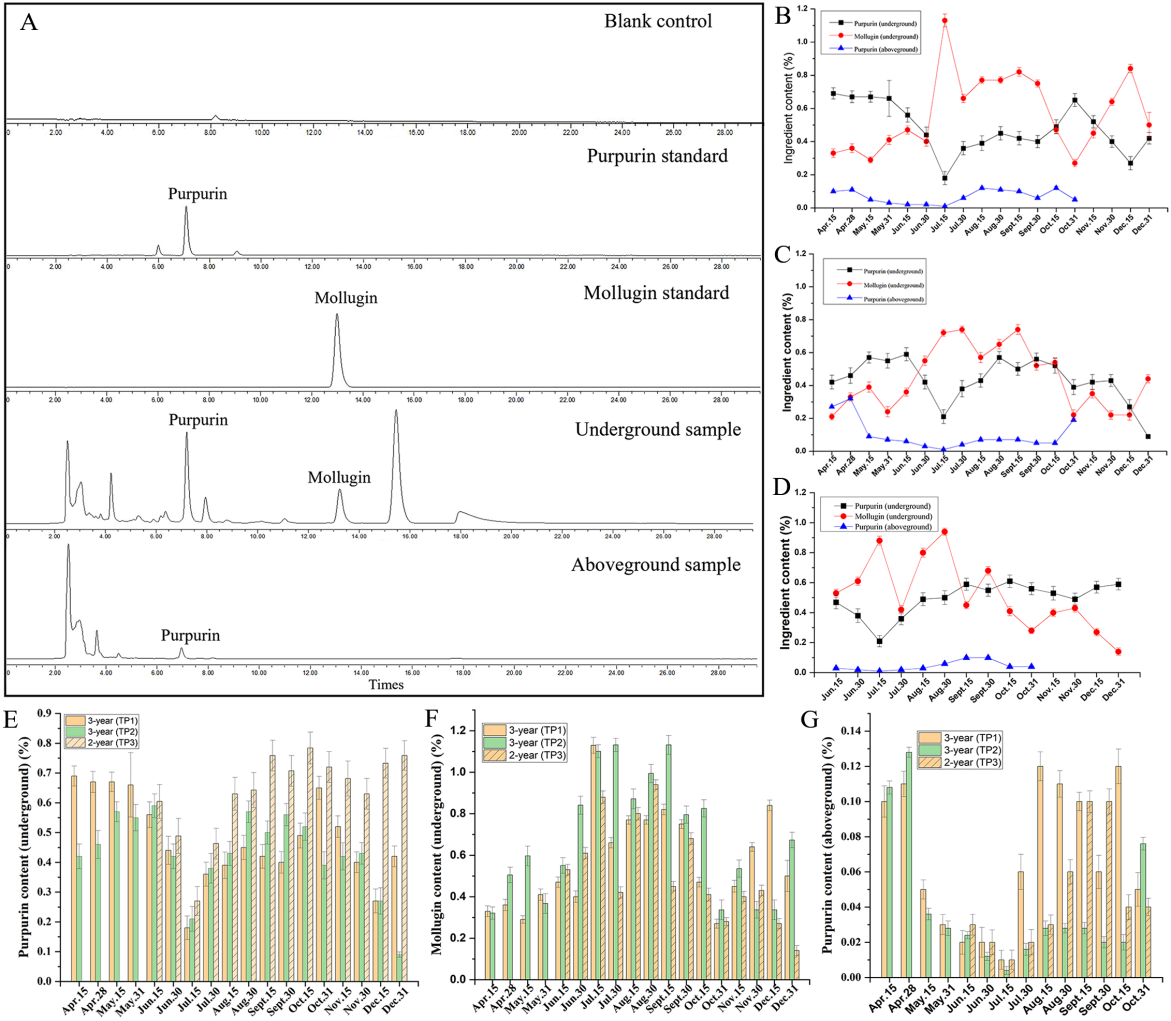
The dynamic accumulation of active ingredients in *R. cordifolia*. **(A)** HPLC chromatograms of mollugin and purpurin from *R. cordifolia* samples. **(B)** Dynamic accumulation of active ingredients in TP1. **(C)** Dynamic accumulation of active ingredients in TP2. **(D)** Dynamic accumulation of active ingredients in TP3. **(E)** Comparative analysis of purpurin content in the underground parts of TP1, TP2, and TP3. **(F)** Comparative analysis of mollugin content in the underground parts of TP1, TP2, and TP3. **(G)** Comparative analysis of purpurin content in the aboveground parts of TP1, TP2, and TP3.

To investigate the dynamic accumulation of active components in *R. cordifolia*, the levels of active ingredient, including mollugin and purpurin, were measured in the development of 3-year-old *R. cordifolia* in TP1 (**Figure 3**). HPLC chromatograms of mollugin and purpurin from *R. cordifolia* samples are shown in **Figure 3A**. As shown in **Figure 3B**, the content of purpurin and mollugin met the standards of the Chinese Pharmacopoeia, except for mollugin in the batches of 31 October, 15 May, and April batches. The dynamic accumulations showed that the purpurin content in the roots exhibited an initially decreasing trend from 15 April to 15 July, with the purpurin content decreasing from 0.69% to 0.18%. It is presumed that this period represents a phase of rapid growth of *R. cordifolia*, during which the energy substances are mainly used for the nutritive growth of the *R. cordifolia*, leading to a gradual decrease in secondary metabolites. Subsequently, the purpurin content showed an increase from 15 July to 31 October, rising from 0.18% to 0.65%. Then, from 15 October to 15 December, the purpurin content decreased to 0.27%, and finally increased to 0.42% on 31 December (**Figure 3B**). Furthermore, the presence of mollugin was also detected. Compared to the purpurin content, the accumulation of mollugin exhibited an opposite trend (**Figure 3B**). From April to July, the mollugin content gradually increased with the rise in ambient temperature during the vegetative growth stage of *R. cordifolia*, reaching a peak of 1.13% in July. Subsequently, on 31 October, the mollugin content level dropped to a low of 0.27%, at which point the aboveground portion of *R. cordifolia* was completely wilted (**Figure 3B**). These phenomena are consistent with the fact that mollugin showed a positive correlation with the carbon content and the carbon-nitrogen ratio of the aerial parts (Wang et al., 2023c). Furthermore, the composition of the aboveground parts of *R. cordifolia* was analyzed. It was found to contain only mollugin, with no trace of purpurin. The pattern of mollugin accumulation mirrored that of the root, but its concentration was notably lower than that of the root (**Figure 3B**).

Similarly, the dynamic accumulation of the main active ingredients in 3-year-old *R. cordifolia*, which were planted in trial plot 2 (TP2) with a much shadier environment, were analyzed (**Figure 3C**). The results indicated that the purpurin content met the standards of the Chinese Pharmacopoeia in all batches, except the 31 December batch. On the other hand, mollugin only conformed to the standards between 30 June and 15 October. The dynamic accumulations of mollugin and purpurin were similar to those of TP1. Furthermore, the active ingredients of *R. cordifolia* in TP1 and TP2 were compared. The total sum contents of purpurin and mollugin were 8.65% and 10.33% in TP1, and 7.79% and 8.00% in TP2, respectively. These results indicate that the growth of *R. cordifolia* in TP1 was better than that in TP2 (**Figures 3E, F**). In addition, the concentration of purpurin was higher in April and May, and lower in July in both the growth periods TP1 and TP2. While the levels of mollugin were higher in July and lower in April for both growth TP1 and TP2, the results also indicated a certain complementary relationship in the accumulation of purpurin and mollugin in *R. cordifolia* (**Figures 3E, F**). The results indicated that cultivated *R. cordifolia* is well-suited for growth in sunny plots, which is more conducive to the accumulation of its primary active ingredients and improves the pharmacodynamic quality of *R. cordifolia*. Combining the results of TP1 and TP2, the harvest period for *R. cordifolia* is from 30 June to 15 October, with the highest quality occurring in August and September.

Based on the market survey, it was found that some *R. cordifolia* plants are harvested biennially. Therefore, the dynamic accumulation of the active ingredients of the biennial *R. cordifolia*, which grows in TP3 under sunny environmental conditions, was also studied from June to December (**Figure 3D**). The results showed that the dynamic accumulations of mollugin and purpurin were similar to those of 3-year-old *R. cordifolia*. The content of purpurin and mollugin can also meet the standards of the Chinese Pharmacopoeia, except for mollugin in batches produced on 31 October and December (**Figure 3D**). These results show that *R. cordifolia* can be harvested every two years. In addition, the total contents of purpurin and mollugin in 3-year-old *R. cordifolia* were 5.96% and 8.94%, respectively, while in 2-year-old *R. cordifolia*, they were 6.90% and 7.24%. The combined content of purpurin and mollugin was 14.91% for 3-year-old *R. cordifolia* and 14.14% for 2-year-old *R. cordifolia*. The analysis reveals that the concentration of active ingredients is slightly higher in the triennial *R. cordifolia* than that in the biennial *R. cordifolia*. Above all, when combined with the yield of *R. cordifolia* (**Figure 2L**), the harvest of 3-year-old *R. cordifolia* plants was better than that of biennial *R. cordifolia*.

In addition, a comprehensive analysis of the dynamic variation in purpurin and mollugin content revealed a specific relationship between the accumulation of purpurin and mollugin. For instance, the purpurin content was highest on 15 Jul and then decreased, while the mollugin content was lowest on 15 Jul and then increased (**Figure 3B**). This suggests a possible transformation relationship in the synthesis process of purpurin and mollugin.

### 3.4 PacBio sequencing and *de novo* assembly of the full-length transcriptome

To obtain full-length transcripts for PacBio long-read sequencing, we created a library by pooling equal amounts of total RNA from four plant tissues: root, stem, leaf, and flower of *R. cordifolia*. A total of 24.27 gigabytes of raw reads were generated, resulting in 249,283 circular consensus sequences (CCS) with 366,366,791 read bases and a mean read length of 1,469 after filtering from the mixed library. This included 83.72% (208,689) of the full-length non-chimeric (FLNC) sequences. Furthermore, a total of 75,157 consensus isoforms with a mean read length of 1,333 were obtained by clustering the FLNC sequences, and 75,139 (99.98%) high-quality consistent sequences were obtained. After eliminating redundant sequences from high-quality isoforms using CD-HIT software, a total of 45,925 non-redundant transcript sequences were obtained for annotation analysis. In addition, the integrity analysis of transcriptome completeness with Benchmarking Universal Single-Copy Orthologs (BUSCO) showed that a total of 303 BUSCO groups were searched. This included 143 (47.2%) complete single-copy BUSCOs, 111 (36.6%) complete duplicated BUSCOs, 18 (5.9%) fragmented BUSCOs, and 31 (10.3%) missing BUSCOs. These results suggest that the transcriptome assembled in our study is relatively complete (**Table 2**). The sequencing data is available at NCBI under the accession number PRJNA1079016.

**Table 2:** Data analysis of the full-length transcriptome in *R. cordifolia*.

Features	Numbers
Read Bases of CCS	366,366,791
CCS Number	249,283
Mean Read Length of CCS	1,469
Number of full-length non-chimeric reads	208,689 (83.72%)
Number of consensus isoforms	75,157
Number of high-quality isoforms	75,139 (99.98%)
Non-redundant transcript sequences	45,925
Complete single-copy BUSCOs	143 (47.2%)
Complete duplicated BUSCOs	111 (36.6%)
Fragmented BUSCOs	18 (5.9%)
Missing BUSCOs	31 (10.3%)

### 3.5 Functional annotation of the transcripts

**Figure 4:**
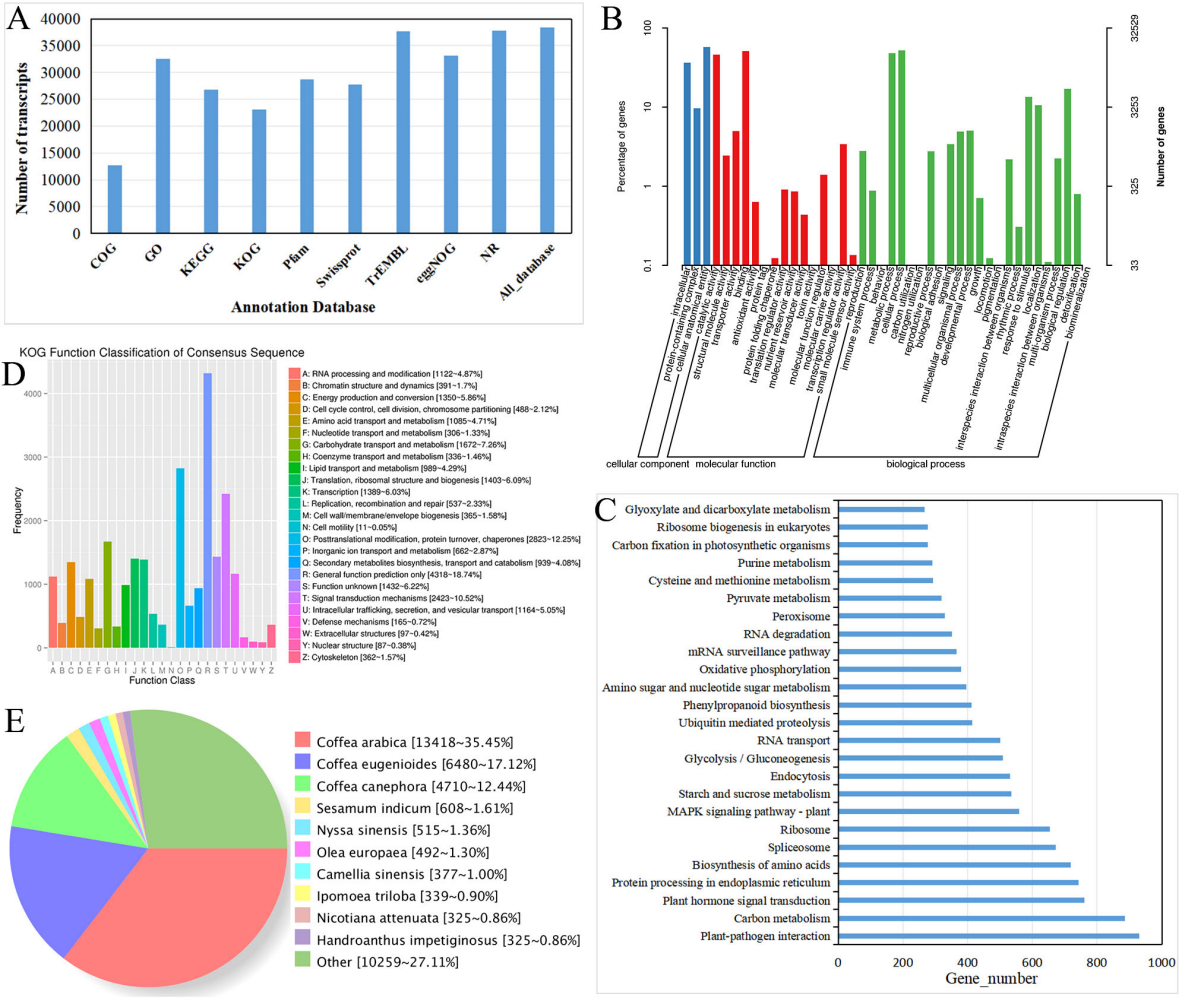
Functional annotation of *R. cordifolia* transcripts. **(A)** Analysis of the full-length transcripts aligned to public databases using histogram diagrams. Histogram diagram analysis of the full-length transcripts aligned to public databases. **(B)** GO enrichment analysis of the transcripts. **(C)** Top 25 KEGG pathway enrichment analysis of the transcripts. **(D)** KOG classification analysis of the transcripts. **(E)** Nr database analysis of the transcripts.

To obtain the most comprehensive annotation for the full-length transcript of *R. cordifolia*, the sequences of the 45,925 non-redundant transcripts were aligned with a series of databases. The results showed that a total of 38,404 (83.62%) non-redundant transcripts were annotated by DIAMOND against nine databases. The potential matches for the GO, KEGG, KOG, COG, Pfam, Swiss-Prot, TrEMBL_Annotation, eggNOG, and NR databases were 32,529 (70.83%), 26,752 (58.25%), 23,037 (50.16%), 12,675 (27.60%), 28,722 (62.54%), 27,740 (60.40%), 37,708 (82.11%), 33,142 (72.17%), and 37,848 (82.41%), respectively (**Supplementary Table 2**; **Figure 4A**).

GO enrichment analysis was utilized to categorize the functions of the full-length transcripts. In total, 32,529 genes were annotated, with three main branches: molecular function (68,305 transcripts), cellular component (87,096 transcripts), and biological process (177,764 transcripts) terms (**Figure 4B**; **Supplementary Table 3**). In the field of biological process, the four largest classifications are cellular processes (17,031), metabolic processes (15,593), biological regulation (5,558), and response to stimuli (4,388), respectively. In terms of molecular function, a significant number of genes were assigned to classes such as binding (16,609), catalytic activity (14,993), transporter activity (1,620), and transcription regulator activity (1,107). In the cellular component, there are 87,096 transcripts, of which 18,655 are related to cellular anatomical entities, 11,828 to intracellular components, and 3,136 to protein-containing complexes.

In the KEGG classification, 2,672 transcripts were annotated in the KEGG database and assigned to 136 biological pathways (**Figure 4C**; **Supplementary Table 4**). The two largest pathways were the metabolic pathways, containing 6,349 transcripts, and the biosynthesis of secondary metabolites, including 3,501 transcripts. In the second level of KEGG, a significant number of genes were assigned to pathways such as Plant-pathogen interaction (ko04626, 930), Carbon metabolism (ko01200, 887), Plant hormone signal transduction (ko04075, 762), Protein processing in the endoplasmic reticulum (ko04141, 742), and Biosynthesis of amino acids (ko01230, 718).

The KOG analysis revealed that 23,037 transcripts were categorized into 26 functional clusters (**Figure 4D**; **Supplementary Table 2**). In addition, the Nr annotation revealed that the homologous species of *R. cordifolia* in the Nr database were *Coffea arabica* (13,418, 35.45%), *Coffea eugenioides* (6,480, 17.12%), and *Coffea canephora* (4,710, 12.44%), all of which belong to the same family, *Rubiaceae* (**Figure 4E**; **Supplementary Table 2**).

### 3.6 Identification of coding sequences (CDS), long non-coding RNAs (LncRNAs), alternative splicing (AS) events, transcription factors (TFs), and simple sequence repeats (SSR)

**Figure 5:**
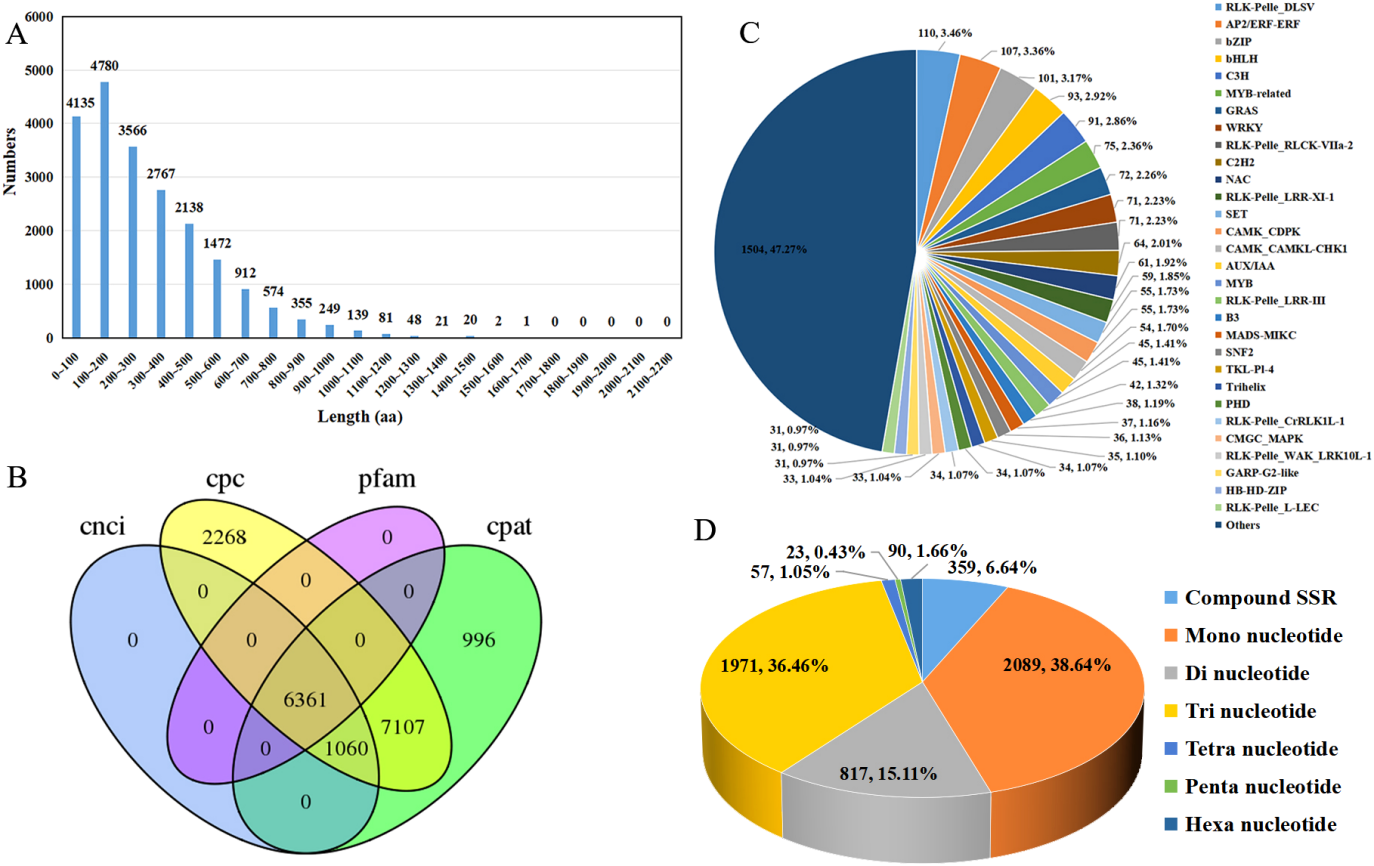
CDS, lncRNAs, transcription factors, and SSR analysis of *R. cordifolia*. **(A)** Length distribution of the amino acid sequences encoded by the complete ORF. **(B)** Venn diagram of lncRNAs identified by CNCI, CPC, CPAT, and Pfam. **(C)** Pie chart analysis of the transcription factor family. **(D)** Pie chart analysis of the SSR types.

To obtain more comprehensive information on transcriptome data, CDS, LncRNAs, AS events, transcription factors, and SSR were analyzed. The TransDecoder software was utilized to predict coding sequences, resulting in a total of 39,678 ORFs, of which 21,260 were complete ORFs. The predicted length of the complete ORF-encoded protein sequence ranges from 0 to 1,700 amino acids, and the distribution of amino acid sequence lengths shows that as the sequence length increases, the number of sequences in the complete ORFs tends to gradually decrease (**Figure 5A**). Furthermore, long non-coding RNAs (lncRNAs) were predicted using the Coding-Non-Coding-Index (CNCI), Coding Potential Calculator (CPAT), Coding Potential Calculator (CPC), and Pfam. In total, 14,528, 15,524, and 45,925 lncRNAs were identified by the CNCI, CPAT, and CPC, respectively. After removing transcripts containing protein domains through Pfam prediction, 6361 lncRNAs were commonly identified by the four methods and were considered the final set of acquired lncRNAs (**Figure 5B**; **Supplementary Table 5**). In addition, a total of 564 AS events were identified (**Supplementary Table 6**). Among them, 444 unigenes had only one AS event, 36 unigenes had two AS events, and 10 unigenes had three AS events. Additionally, tissues_transcript_34052, tissues_transcript_63033, and tissues_transcript_3265 had 8, 6, and 4 AS events, respectively. Furthermore, the type of these alternative splicing events cannot be identified because the reference genome is not included.

TFs are crucial regulators of gene expression and play significant roles in plant growth and development. A total of 3182 TFs were identified by using iTAK software, including 1316 protein kinases (PK) and 484 transcriptional regulators (TR). Among them, 199 families were annotated, including RLK-Pelle_DLSV (110, 3.46%), AP2/ERF-ERF (107, 3.36%), bZIP (101, 3.17%), bHLH (93, 2.92%), C3H (91, 2.86%), MYB-related (75, 2.36%), and GRAS (72, 2.26%) (**Figure 5C**). In addition, the full-length transcripts of *R. cordifolia* provide important resources for the development of SSRs. A total of 5,406 SSRs were identified from 4,654 transcripts using the MISA Perl script, and 590 of these transcripts contained more than one type of SSR. For the types of SSRs, mononucleotide repeats (2089, 38.64%) were the most abundant, followed by trinucleotide repeats (1971, 36.46%), dinucleotide repeats (817, 15.11%), compound SSRs (359, 6.64%), hexanucleotide (90, 1.66%), and tetranucleotide (57, 1.05%). Penta-nucleotide repeats were the least frequent (23, 0.43%) (**Figure 5D**; **Supplementary Table 7**).

### 3.7 Identification of full-length transcripts potentially involved in anthraquinone biosynthesis

**Figure 6:**
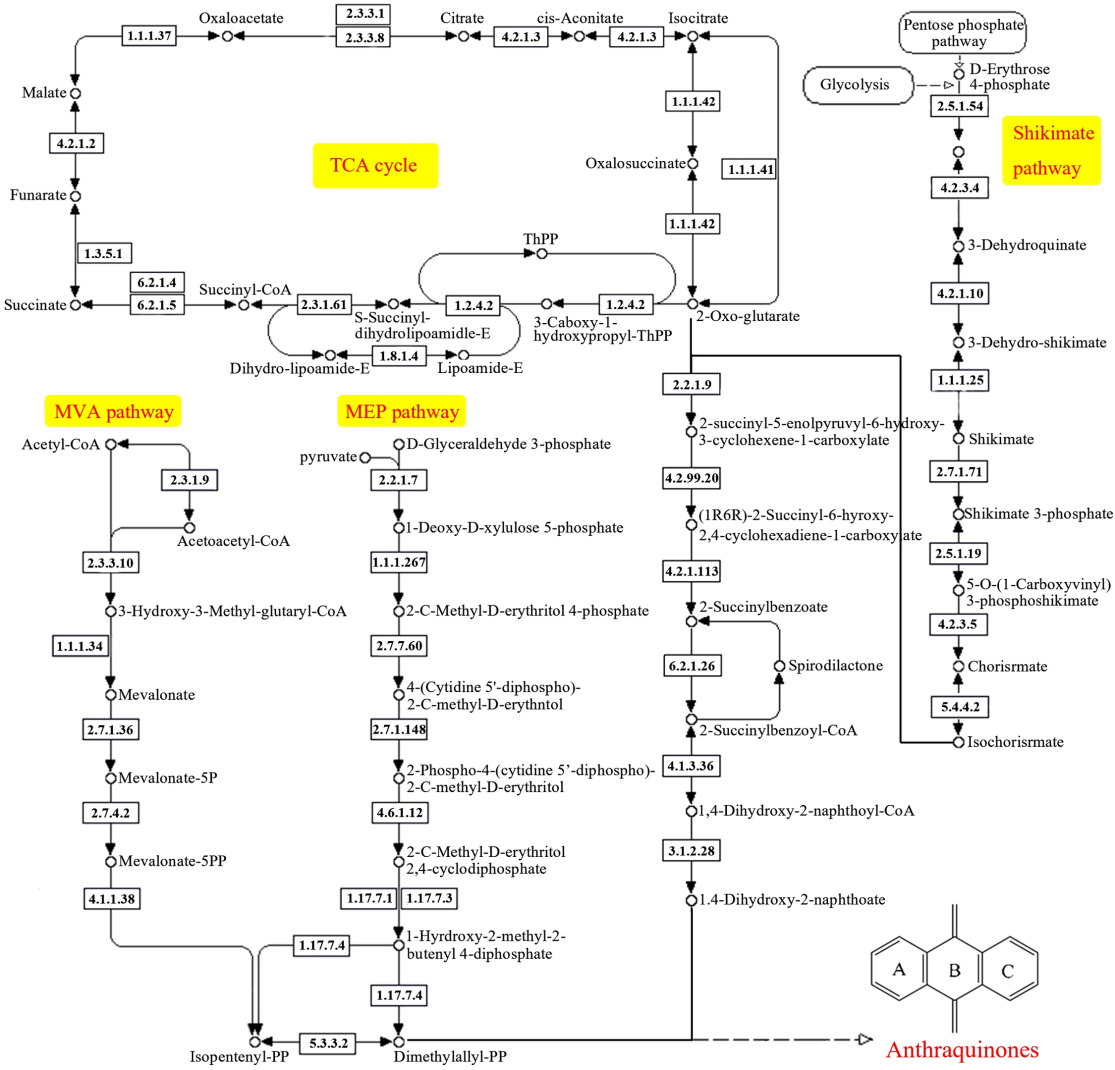
Illustration of the anthraquinone biosynthesis pathway in *R. cordifolia*.

Currently, it is widely believed that anthraquinones are formed through the shikimic acid/o-succinylbenzoic acid pathway in *R. cordifolia*. The synthesis of anthraquinone from shikimic acid/o-succinylbenzoic acid pathway precursors involves a variety of metabolic pathways, mainly including the shikimic acid pathway, TCA cycle, MVA pathway, and MEP pathway (Liang et al., 2020). Based on these metabolic pathways, we constructed an anthraquinone biosynthesis pathway in *R. cordifolia* in our study (**Figure 6**). In total, 280 unigenes were identified as being involved in the formation of anthraquinones in *R. cordifolia* (**Figure 6**, **Table 3**; **Supplementary Table 8**). In the TCA cycle, 128 unigenes were identified, including malate dehydrogenase (17 unigenes), citrate synthase (10 unigenes), ATP citrate (pro-S)-lyase (16 unigenes), and aconitate hydratase (12 unigenes). Among these, 2-oxoglutarate is an important substrate for anthraquinone synthesis through the shikimic acid/o-succinylbenzoic acid pathway. Increasing 2-oxoglutarate levels can enhance the biosynthesis of anthraquinones (Zhang et al., 2009). Additionally, isocitrate dehydrogenase (15 unigenes) and isocitrate dehydrogenase (NAD+) (11 unigenes) may also play a significant role in regulating anthraquinone biosynthesis. In the MVA pathway, six enzymes and 24 unigenes are involved, including *AACT* (4 unigenes), *HMGS* (2 unigenes), *HMGR* (8 unigenes), *MVK* (3 unigenes), *PMK* (2 unigenes), and *MVD* (3 unigenes). In the MEP pathway, eight enzymes and 44 unigenes are involved, including *DXS* (13 unigenes), *DXR* (10 unigenes), *ispD*/*MCT* (1 unigene), *ispE*/*CMK* (2 unigenes), *ispF*/*MDS* (2 unigenes), *ispG*/*HDS* (3 unigenes), *ispH*/*HDR* (9 unigenes), and *IDI*/*IPPs* (4 unigenes). In addition, 11 enzymes and 76 unigenes in the shikimate pathway were also involved. These include *aroF* (13 unigenes), *aroB* (3 unigenes), *aroDE* (13 unigenes), *aroK* (8 unigenes), *aroA* (5 unigenes), *aroC* (5 unigenes), *menF* (2 unigenes), *menC/D/H* (8 unigenes), *menE* (7 unigenes), *menB* (8 unigenes), and *menI* (4 unigenes).

**Table 3:** Unigenes involved in anthraquinone biosynthesis in *R. cordifolia*.

Pathway	EC	KEGG ID	Gene name	Enzyme Symbol	No. of unigenes
MVA pathway	2.3.1.9	K00626	acetyl-CoA C-acetyltransferase	AACT	4
	2.3.3.10	K01641	hydroxymethylglutaryl-CoA synthase	HMGS	2
	1.1.1.34	K00021	hydroxymethylglutaryl-CoA reductase	HMGR	8
	2.7.1.36	K00869	mevalonate kinase	MVK	3
	2.7.4.2	K00938	phosphomevalonate kinase	PMK	2
	4.1.1.33	K01597	diphosphomevalonate decarboxylase	MVD	3
MEP pathway	2.2.1.7	K01662	1-deoxy-D-xylulose-5-phosphate synthase	DXS	13
	1.1.1.267	K00099	1-deoxy-D-xylulose-5-phosphate reductoisomerase	DXR	10
	2.7.7.60	K00991	2-C-methyl-D-erythritol 4-phosphate cytidylyltransferase	ispD/MCT	1
	2.7.1.148	K00919	4-diphosphocytidyl-2-C-methyl-D-erythritol kinase	ispE/CMK	2
	4.6.1.12	K01770	2-C-methyl-D-erythritol 2,4-cyclodiphosphate synthase	ispF/MDS	2
	1.17.7.1/1.17.7.3	K03526	(E)-4-hydroxy-3-methylbut-2-enyl-diphosphate synthase	ispG/HDS	3
	1.17.7.4	K03527	4-hydroxy-3-methylbut-2-en-1-yl diphosphate reductase	ispH/HDR	9
	5.3.3.2	K01823	isopentenyl-diphosphate Delta-isomerase	IDI	4
TCA cycle	1.1.1.37	K00026	malate dehydrogenase	MDH2	17
	2.3.3.1	K01647	citrate synthase	CS/gltA	10
	2.3.3.8	K01648	ATP citrate (pro-S)-lyase	ACLY	16
	4.2.1.3	K01681	aconitate hydratase	ACO/acnA	12
	1.1.1.42	K00031	isocitrate dehydrogenase	IDH1/icd	15
	1.1.1.41	K00030	isocitrate dehydrogenase (NAD+)	IDH3	11
	1.2.4.2	K00164	2-oxoglutarate dehydrogenase E1 component	OGDH/sucA	5
	1.8.1.4	K00382	dihydrolipoyl dehydrogenase	DLD/lpd/pdhD	5
	2.3.1.61	K00658	2-oxoglutarate dehydrogenase E2 component (dihydrolipoamide succinyltransferase)	DLST/sucB	7
	6.2.1.4/6.2.1.5	K01899	succinyl-CoA synthetase alpha subunit	LSC1	2
		K01900	succinyl-CoA synthetase beta subunit	LSC2	2
	1.3.5.1	K00234	succinate dehydrogenase (ubiquinone) flavoprotein subunit	SDHA/SDH1	18
		K00236	succinate dehydrogenase (ubiquinone) cytochrome b560 subunit	SDHC/SDH3	5
		K00235	succinate dehydrogenase (ubiquinone) iron-sulfur subunit	SDHB/SDH2	4
	4.2.1.2	K01679	fumarate hydratase, class II	fumC/FH	9
Shikimate pathway	2.5.1.54	K01626	3-deoxy-7-phosphoheptulonate synthase	aroF/aroG/aroH	13
	4.2.3.4	K01735	3-dehydroquinate synthase	aroB	3
	4.2.1.10/1.1.1.25	K13832	3-dehydroquinate dehydratase/shikimate dehydrogenase	aroDE/DHQ-SDH	13
	2.7.1.71	K00891	shikimate kinase	aroK/aroL	8
	2.5.1.19	K00800	3-phosphoshikimate 1-carboxyvinyltransferase	aroA	5
	4.2.3.5	K01736	chorismate synthase	aroC	5
	5.4.4.2	K02552	menaquinone-specific isochorismate synthase	menF	2
	5.4.4.2/2.2.1.9/4.2.99.20/4.2.1.113	K14759	isochorismate synthase/2-succinyl-5-enolpyruvyl-6-hydroxy-3- cyclohexene-1-carboxylate synthase/2-succinyl-6-hydroxy-2,4- cyclohexadiene-1-carboxylate synthase/o-succinylbenzoate synthase	menC/D/H	8
	6.2.1.26	K14760	o-succinylbenzoate-CoA ligase	menE	7
	4.1.3.36	K01661	naphthoate synthase	menB	8
	3.1.2.28	K19222	1,4-dihydroxy-2-naphthoyl-CoA hydrolase	menI/DHNAT	4

### 3.8 RT-qPCR analysis of full-length transcripts potentially involved in anthraquinone biosynthesis

**Figure 7:**
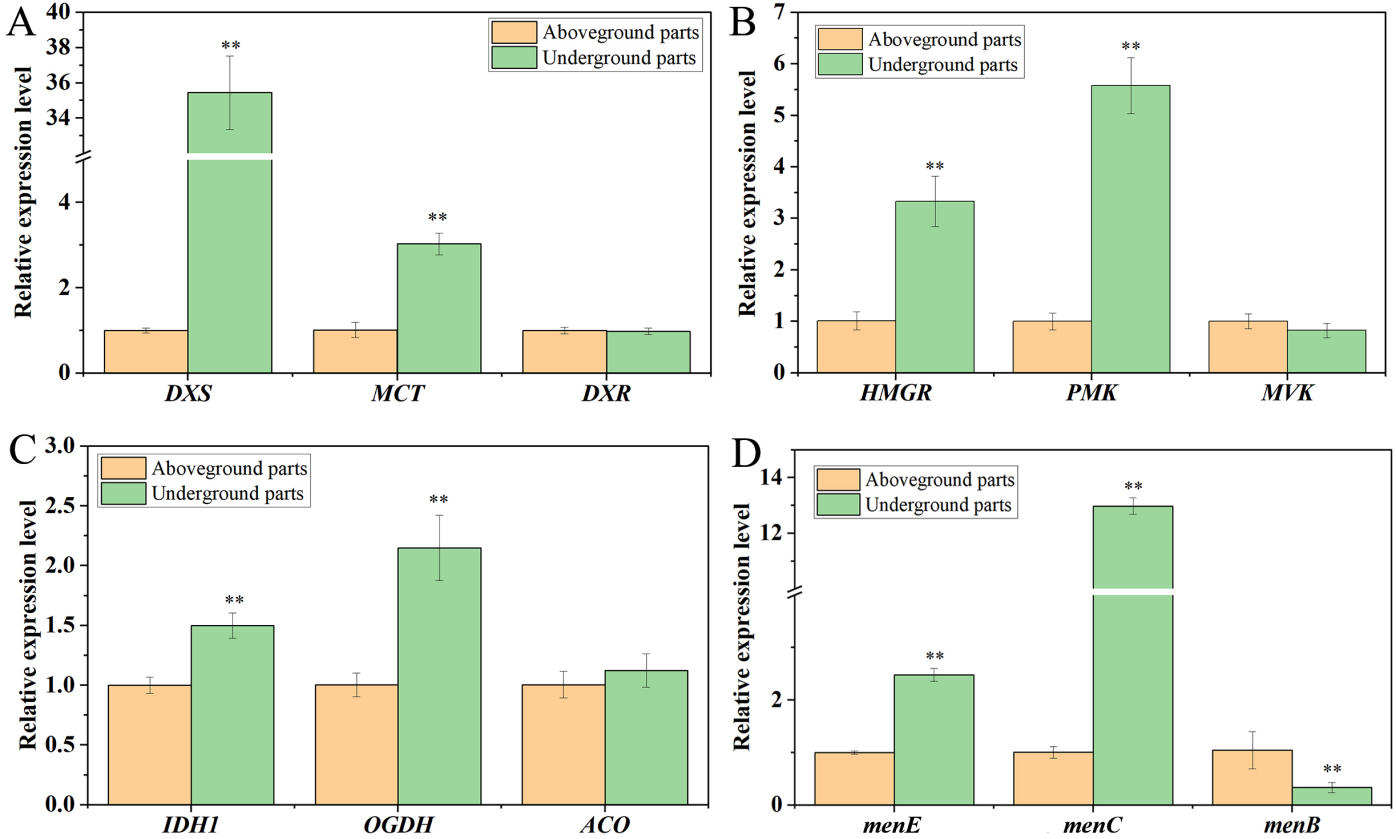
RT-qPCR analysis of 12 genes potentially involved in anthraquinone biosynthesis in the underground and aboveground parts of *R. cordifolia*. **(A)** Expression profiles of three MVA pathway genes; **(B)** Expression profiles of three MEP pathway genes; **(C)** Expression profiles of three TCA cycle pathway genes; **(D)** Expression profiles of three shikimate acid pathway genes. Each set had three replicates. “**” indicates statistically significant differences between different parts (P < 0.01, Duncan test).

To validate the quality of our putative results of full-length transcripts involved in anthraquinone biosynthesis, 12 candidate genes related to anthraquinone biosynthesis were randomly selected for expression analysis using the qRT-PCR method. These genes included MVA pathway genes (*HMGR*, *MVK*, and *PMK*), MEP pathway genes (*DXS*, *DXR*, and *MCT*), TCA cycle genes (*ACO*, *IDH1*, *OGDH*), and shikimic acid pathway genes (*menC/D/H*, *menE*, and *menB*) (**Figure 7**). The results showed that the expression levels of 9 out of 12 genes were significantly different in aboveground parts compared to underground parts. To validate the quality of our putative results of full-length transcripts involved in anthraquinone biosynthesis, 12 candidate genes related to anthraquinone biosynthesis were randomly selected for expression analysis using the qRT-PCR method. These genes include those involved in the MVA pathway (*HMGR*, *MVK*, and *PMK*), MEP pathway (*DXS*, *DXR*, and *MCT*), TCA cycle (*ACO*, *IDH1*, *OGDH*), and shikimic acid pathway (*menC/D/H*, *menE*, and *menB*) (**Figure 7**). The results showed that the expression levels of 9 out of 12 genes were significantly different in aboveground parts compared to underground parts.

In comparing underground and aboveground parts, the relative expression of the *HMGR* and *PMK* genes in the MVA pathway significantly increased by up to 3.33- and 5.58-fold, respectively. The *DXS* and *MCT* genes in the MEP pathway also showed significant increases, up to 35.44- and 3.03-fold, respectively. Additionally, the *menE* and *menC/D/H* genes in the shikimic acid pathway exhibited significant increases, up to 2.48- and 12.98-fold, respectively. The *IDH1* and *OGDH* genes in the TCA cycle pathway were also significantly increased, up to 1.50- and 2.15-fold, respectively. In contrast, the expression of the *menB* gene decreased, and there was no difference in the expression of the other 3 genes, including *MVK*, *DXR*, and *ACO*. These findings indicate the presence of functional differentiation among different members of the same class of genes or gene family.

## 4 Discussion

### 4.1 Dynamic growth characteristics and accumulation of active ingredients in *R. cordifolia*

*R. cordifolia* is a type of Chinese herbal plant that can be used orally or topically. With the ongoing research and development in modern medicine, the use of *R. cordifolia* is becoming increasingly widespread, and its market demand is continuously increasing (Wen et al., 2022). Traditional Chinese medicinal materials are mainly collected from the wild. However, due to the ongoing depletion of wild resources, the reserves of wild herbs are unable to meet market demand. Therefore, it is crucial to prioritize the artificial cultivation of Chinese medicinal materials. At present, the majority of *R. cordifolia* on the market is still harvested from wild resources. On the one hand, the wild resources are insufficient, even though the area where *R. cordifolia* is produced is extensive. Additionally, the recovery ability of *R. cordifolia* is very slow after one-time mining, taking several years to recover. On the other hand, the mining of *R. cordifolia* in the wild environment is difficult, leading to increased labor costs and a decrease in mining personnel (Wang et al., 2023c). Therefore, it is necessary to conduct research on the cultivation technology of *R. cordifolia*. Currently, the artificial cultivation technology of *R. cordifolia* is still in the exploratory stage, and there is still no comprehensive scientific guidance for its cultivation, growth characteristics, and the dynamic accumulation of main active ingredients. In this study, we investigated the growth characteristics and the dynamic accumulation of the main active ingredients. The phenological period of cultivated *R. cordifolia* was from mid-March to mid-December, exhibiting similarities to that of wild growth. *R. cordifolia* thrives in open forests, forest margins, shrubs, or grasslands, as it prefers a semi-shaded environment for its wild growth. The study found that the biomass and secondary metabolites of *R. cordifolia* were greater in the sunny environment compared to the shady environment. These results demonstrate that adequate sunlight promotes the accumulation of biomass and secondary metabolites of *R. cordifolia*.

Previous studies have shown that the accumulation of secondary metabolites changes dynamically as plants grow, and the quality of Chinese medicinal materials depends on the concentration of secondary metabolites. Therefore, the collection of medicinal materials is time-sensitive, and the quality of materials collected at different times varies. This can lead to substandard quality, which ultimately affects the therapeutic efficacy of the medicinal materials. It is generally believed that root and rhizome herbs are harvested in the autumn or winter when the above-ground part is about to wither, or in the early spring before germination, or at the beginning of sprouting. In the history of materia medica records, the harvesting period for *R. cordifolia* is not consistent. The “Compendium of Materia Medica” records it as being harvested from February to March; the “Chinese Materia Medica” records that *R. cordifolia* was traditionally harvested in August, but according to the Chinese Pharmacopeia (Committee, 2020), it is now usually considered to be harvested in spring or autumn. It is evident that there is still uncertainty about the harvesting time of *R. cordifolia*. In our study, we analyzed the annual accumulation of the main active ingredients, purpurin and mollugin, prescribed by the Chinese Pharmacopoeia in *R. cordifolia*. The results indicated that both main active ingredients met the standards of the Chinese Pharmacopoeia from 30 June to 15 October, with higher quality observed in August and September. These findings are consistent with previous reports by Wang et al (Wang et al., 2023c). Based on the results of our study and previous reports, it can be concluded that the appropriate harvesting period for *R. cordifolia* is from 30 June to 15 October, with August being the optimal time.

In addition, the quality of perennial Chinese medicinal materials is closely related to the growth years of plants. The harvest years of many Chinese medicinal materials are clearly specified in “The Production Quality Management Standards of Chinese Medicinal Materials”, which also emphasizes the importance of growth years to the quality of some medicinal materials (Zhang et al., 2010). Therefore, the harvest time for herbs also depends on their growth stage. For *Panax ginseng*, it is better to choose *P. ginseng* that has grown for more than five years (Shan et al., 2014). For *Paris Polyphylla* var. Yunnanensis, the optimal harvest time is recommended at the 7th or 8th year (Wang and Li, 2018). The best harvest time for *Panax Notoginseng* is December, and the optimal harvest time is 3 years after planting (Cun et al., 2022). *R. cordifolia* was primarily harvested from the wild, and the year of harvest could not be determined. As a result, some *R. cordifolia* that had not reached the appropriate age for use have also entered the market, leading to an overall decline in the quality of *R. cordifolia*. It was generally believed that the quality of *R. cordifolia* was better when the plant was three years old or older. It has also been reported that *R. cordifolia* can be harvested after two years. Therefore, our study further examined the quality of biennial and triennial *R. cordifolia*. The results showed that the main ingredients of biennials could also meet the standards of the Chinese Pharmacopoeia from 30 June to 15 October. These results indicate that biennial *R. cordifolia* could also be harvested, and the decision to harvest or not could be based on the current year’s price of *R. cordifolia*.

Furthermore, the harvesting time for medicinal materials is typically determined by the goal of optimizing quality and maximizing yield. For instance, the gentiopicroside content of radix Gentianae macrophyllae is highest when the plant is 3 years old. However, the yield is low at this stage. In contrast, the gentiopicroside content is slightly reduced in the 4-year-old plant, but the yield increases significantly and the overall quality of the plant is also improved. Therefore, the most appropriate harvest time for radix Gentianae macrophyllae is determined to be 4 years (Chen et al., 2010). In *Achyranthes bidentata*, the contents of organic chemical components (β-ecdysterone, 25R-hyssopterone, 25S-hyssopterone, oleanolic acid) with different growth years (2~6 years) and harvesting periods (September to December) from April 2015 to December 2021 were detected, and the comprehensive results suggested that mid-November of the fourth year is the best time to harvest *Achyranthes bidentata* (Gao, 2024). In our study, the comparison of yield and main ingredient content in the underground parts of biennials and triennials revealed that the content of triennials was not significantly different from that of biennials, but the yield increased significantly. Therefore, while biennial harvesting shortens the growing season and reduces overall yield and quality, it is recommended to harvest *R. cordifolia* at 3 years of age.

### 4.2 Mining anthraquinone biosynthesis genes in *R. cordifolia*

Anthraquinone and its derivatives are very important secondary metabolites in plants, and play many functions involved in plant growth and development, such as photoprotection, improving plant disease resistance, and acting as the main active ingredients for medicinal effects (Dey et al., 2022; Martínez-Romero et al., 2013; Nain-Perez et al., 2022). Anthraquinones in higher plants are primarily found in *Polygonaceae*, *Leguminosae*, *Rhamnaceae*, *Rubiaceae*, and *Xanthorrhoeaceae* (Dave and Ledwani, 2012; Liang et al., 2020). Among these, the anthraquinone metabolites of certain plants exhibit significant pharmacological activities. For example, five anthraquinones in rhubarb, such as rhein, emodin, chrysophanol, emodin methyl ether, and aloe emodin, are the primary medicinal components of rhubarb. They have antibacterial, anti-inflammatory, and anti-cancer properties (Pandith et al., 2018). In *R. cordifolia*, one of the main medicinal ingredients is purpurin, which belongs to the anthraquinone component. It shows antigenotoxic, anticancer, neuromodulatory, and antimicrobial potential associated with antioxidant action in *in vivo* and *in vitro* experiments (Singh et al., 2021). The *in vivo* synthesis process of anthraquinones is highly complex, involving multiple metabolites from various metabolic pathways. At present, the biosynthesis pathway of anthraquinones is not fully understood. It is generally believed that the biosynthesis of anthraquinones in plants mainly involves two metabolic pathways: the shikimate/o-succinylbenzoic acid pathway and the polyketide pathway (Zhou et al., 2021). Previous reports have shown that the shikimic acid/o-succinylbenzoic acid pathway for anthraquinone biosynthesis mainly exists in *Rubiaceae* plants (Wang JH et al., 2021). The precursors of the anthraquinone nucleus, synthesized by the shikimic acid/o-succinylbenzoic acid pathway, originate from a variety of metabolic pathways, including the shikimic acid pathway, the TCA cycle, the MVA pathway, and the MEP pathway (Liang et al., 2020). At present, with the continuous exploration of the pharmacodynamic activity of anthraquinones, more and more species of anthraquinones biosynthesis genes have been reported (Wang et al., 2023a). *Rheum tanguticum* genome assembly provides valuable insights into the genetic bases of anthraquinone biosynthesis (Li Y et al., 2023). In *Polygonum cuspidatum*, anthraquinone biosynthesis candidate genes were analyzed by tissue-specific transcriptome (Wang X et al., 2021). And putative genes involved in anthraquinone biosynthesis analyzed by *de novo* transcriptome in *Rubia yunnanensis* (Zhang et al., 2022). In this study, a total of 280 unigenes involved in anthraquinone synthesis were identified by SMRT technology based on the metabolic pathway of anthraquinone synthesis in *R. cordifolia*, including 128 unigenes in the TCA cycle, 24 unigenes in the MVA pathway, 44 unigenes in the MEP pathway, and 76 unigenes in the shikimate pathway.

In the mevalonate (MVA) and methylerythritol phosphate (MEP) pathways, it was found that mevalonate kinase (MVK) and HMG-CoA reductase are key enzymes in regulating the MVA pathway, and the MEP pathway providing the prenyl (isoprenoid) chain by regulating the biosynthesis of anthraquinone (Liu et al., 2024). In the *Morinda citrifolia* cell line, overexpression of the *DXS* gene induced a 24% increase in anthraquinone production, indicating that *DXS* has a significant effect on anthraquinone biosynthesis (Quevedo et al., 2010). In this study, the expression of *HMGR*, *PMK*, *DXS*, and *MCT* genes in *R. cordifolia* was analyzed. The results indicated that the expression levels of these genes in the underground part were higher than those in the above-ground part, which was consistent with the distribution of anthraquinone content in *R. cordifolia*. These results were similar to the pattern of gene expression in *R. yunnanensis*, which also showed higher expression in roots. (Zhang et al., 2022). These results showed that the *HMGR*, *PMK*, *DXS*, and *MCT* genes were mainly expressed in the roots, and might play important regulatory roles in the synthesis of anthraquinone in *R. cordifolia*.

Furthermore, α-ketoglutarate (also known as 2-oxoglutarate) is a crucial precursor for anthraquinone synthesis via the shikimic acid/o-succinylbenzoic acid pathway (Murthy et al., 2023). In addition, isochorismic acid (IC) is a product of the shikimic acid pathway and acts as an important substrate in anthraquinone metabolism and synthesis (Kaiser and Leistner, 1992). Isochorismate synthase (ICS) is a crucial enzyme that catalyzes the conversion of chorismic acid (CHA) to IC in the anthraquinone pathway and plays a significant role in the regulation of anthraquinone synthesis (Wang et al., 2023a). It has been reported that the production of anthraquinone in the cell culture medium of *Morinda citrifolia* consistently increased with the rise in ICS activity (Stalman et al., 2003). In our study, we also analyzed the expression levels of the *MCT*, *ACO*, *IDH1*, *OGDH*, *menC/D/H*, *menE*, and *menB* genes. The results indicated that the expression levels of *menE*, *menC/D/H*, *IDH1*, and *OGDH* genes were higher in the underground part than in the above-ground part. This suggests that these genes may play a positive role in regulating anthraquinone biosynthesis in *R. cordifolia*. In future studies, we will carry out functional studies around these genes, and further confirm their roles in anthraquinone biosynthesis through enzyme activity assays, gene overexpression, or gene silencing techniques. In addition, we will use multi-omics technology to explore more structural genes and regulatory genes related to anthraquinone synthesis, so as to improve the gene regulatory network of anthraquinone biosynthesis. Above all, the identification of these candidate genes is crucial for the subsequent functional verification of related genes and lays an important foundation for further research on anthraquinone biosynthesis in *R. cordifolia*.

## 5 Conclusion

In this study, we integrate research on the dynamics of growth characteristics, the dynamic accumulation of secondary metabolites, and the identification of important genes related to active ingredients in cultivated *R. cordifolia*. The results of the dynamic growth characteristics and secondary metabolite accumulation studies indicated that the growth of plants and the accumulation of secondary metabolites, as well as the quality of medicinal materials, were affected by different growth environments and years. Among them, the growth quality of *R. cordifolia* is better in a sunny environment than in shady environment. Triennial *R. cordifolia* is better than biennial, and the dynamic accumulation of purpurin and mollugin indicates that the optimal harvesting period for *R. cordifolia* should be between 30 June and 15 October. Additionally, the underground parts, including roots and rhizomes, have potential as medicinal parts. Taken together, these results provide significant implications for the management of cultivation and harvesting of cultivated *R. cordifolia*. Furthermore, for the first time, single-molecule real-time (SMRT) sequencing has provided a comprehensive understanding of anthraquinone biosynthesis in *R. cordifolia*. A total of 45,925 full-length transcripts, 564 alternative splicing (AS) events, 3182 transcription factors (TFs), 6454 SSRs, and 6361 lncRNAs were identified. In addition, a biosynthetic pathway for anthraquinones involving 280 full-length transcripts was proposed. This discovery has the potential to accelerate the progress of synthetic biology for purpurin and mollugin, as well as the engineering of *R. cordifolia*.

## Data Availability

The datasets presented in this study can be found in online repositories. The names of the repository/repositories and accession number(s) can be found below: https://www.ncbi.nlm.nih.gov/, PRJNA1079016.
